# Representation of $(p,q)$-Bernstein polynomials in terms of $(p,q)$-Jacobi polynomials

**DOI:** 10.1186/s13660-017-1443-7

**Published:** 2017-07-17

**Authors:** F Soleyman, I Area, M Masjed-Jamei, JJ Nieto

**Affiliations:** 1 Department of Mathematics, K. N. Toosi University of Technology, P.O. Box 16315-1618, Tehran, Iran; 2 Departamento de Matemática Aplicada II, E.E. Aeronáutica e do Espazo, Universidade de Vigo, Campus As Lagoas s/n, Ourense, 32004 Spain; 3 Facultade de Matemáticas, Universidade de Santiago de Compostela, Santiago de Compostela, 15782 Spain

**Keywords:** 34B24, 39A70, $(p,q)$-Bernstein polynoimals, $(p,q)$-Pearson difference equation, $(p,q)$-orthogonal solutions, $(p,q)$-difference operator

## Abstract

A representation of $(p,q)$-Bernstein polynomials in terms of $(p,q)$-Jacobi polynomials is obtained.

## Introduction

Classical univariate Bernstein polynomials were introduced by Bernstein in a constructive proof for the Stone-Weierstrass approximation theorem [1], and they are defined as [2]

$$b_{i}^{n}(x)=\binom{n}{i} x^{i}(1-x)^{n-i}, \quad i=0,1,\dots,n. $$

They form a basis of polynomials and satisfy a number of important properties as non-negativity ($b_{i}^{n}(x) \geq0$ for $0 \leq x \leq 1$), partition of unity ($\sum_{i=0}^{n} b_{i}^{n}(x)=1$) or symmetry ($b_{i}^{n}(x)=b_{n-i}^{n}(1-x)$).

For a given real-valued defined and bounded function *f* on the interval $[0,1]$, the *n*th Bernstein polynomial for *f* is

$$B_{n}(f) (x)=\sum_{k=0}^{n} b_{k}^{n}(x) f \biggl(\frac{k}{n} \biggr). $$

Then, for each point *x* of continuity of *f*, we have $B_{n}(f)(x) \to f(x)$ as $n \to\infty$. Moreover, if *f* is continuous on $[0,1]$ then $B_{n}(f)$ converges uniformly to *f* as $n \to\infty$. Also, for each point *x* of differentiability of *f*, we have $B_{n}'(f)(x) \to f'(x)$ as $n \to\infty$ and if *f* is continuously differentiable on $[0,1]$ then $B_{n}'(f)$ converges to $f'$ uniformly as $n \to \infty$.

Bernstein polynomials have been generalized in the framework of *q*-calculus. More precisely, Lupaş [3] initiated the application of *q*-calculus in area of the approximation theory, and introduced the *q*-Bernstein polynomials. Later on, Philips [4] proposed and studied other *q*-Bernstein polynomials. In both the classical case and in its *q*-analogs, expansions of Bernstein polynomials have been obtained in terms of appropriate orthogonal bases [5, 6].

Mursaleen *et al.* [7] recently introduced first the concept of $(p, q)$-calculus in approximation theory and studied the $(p, q)$-analog of Bernstein operators. The approximation properties for these operators based on Korovkin’s theorem and some direct theorems were considered [8]. Also, many well-known approximation operators have been introduced using these techniques, such as Bleimann-Butzer-Hahn operators [9] and Szász-Mirakyan operators [10]. Very recently Milovanović *et al.* [11] considered a $(p, q)$-analog of the beta operators and using it proposed an integral modification of the generalized Bernstein polynomials. $(p,q)$-analogs of classical orthogonal polynomials have been characterized in [12].

The main aim of this work is to obtain a representation of $(p,q)$-Bernstein polynomials in terms of suitable $(p,q)$-orthogonal polynomials, where the connection coefficients are proved to satisfy a three-term recurrence relation. For this purpose, we have divided the work in two sections. First, we present the basic definitions and notations. Later, in Section 3 we obtain the main results of this work relating $(p,q)$-Bernstein polynomials and $(p,q)$-Jacobi orthogonal polynomials.

## Basic definitions and notations

Next, we summarize the basic definitions and results which can be found in [13–18] and the references therein.

The $(p,q)$-power is defined as

1 $$ \bigl((a,b);(p,q)\bigr)_{k}=\prod _{j=0}^{k-1} \bigl(ap^{j}-bq^{j} \bigr)\quad\text{with } \bigl((a,b);(p,q)\bigr)_{0}=1. $$

The $(p,q)$-hypergeometric series is defined as

2 $$ \begin{aligned}[b] & {}_{r} \Phi_{s}\left ( \textstyle\begin{array}{c}{(a_{1p},a_{1q}),\dots,(a_{rp},a_{rq})}\\ {(b_{1p},b_{1q}),\dots,(b_{sp},b_{sq})} \end{array}\displaystyle \Big|{(p,q)};{z} \right ) \\ &\quad= \sum_{j=0}^{\infty}\frac{((a_{1p},a_{1q}),\dots,(a_{rp},a_{rq});(p, q))_{j}}{((b_{1p},b_{1q}),\dots,(b_{sp},b_{sq}); (p,q))_{j}} \frac {z^{j}}{((p,q);(p,q))_{j}} \bigl((-1)^{j} (q/p)^{\frac{j(j-1)}{2}} \bigr)^{1+s-r}, \end{aligned} $$

where

$$\bigl((a_{1p},a_{1q}),\dots,(a_{rp},a_{rq});(p, q) \bigr)_{j}=\prod_{s=1}^{r} \bigl((a_{sp},a_{sq});(p, q) \bigr)_{j}, $$

and $r, s \in \mathbb {Z}_{+}$ and $a_{1p},a_{1q},\dots ,a_{rp},a_{rq},b_{1p},b_{1q},\dots,b_{sp},b_{sq},z \in \mathbb {C}$.

The $(p,q)$-difference operator is defined as (see *e.g.* [14])

3 $$ (\text{${\mathcal {D}}_{p,q}$}f) (x)=\frac{\text{${\mathcal {L}}_{p}$} f(x)-\text{${\mathcal {L}}_{q}$} f(x)}{(p-q)x},\quad x\neq0, $$

where the shift operator is defined by

4 $$ \text{${\mathcal {L}}_{a}$}h(x)=h(ax), $$

and $(\text{${\mathcal {D}}_{p,q}$}f)(0)=f'(0)$, provided that *f* is differentiable at 0.

The $(p,q)$-Bernstein polynomials are defined as

5 $$ b_{i}^{n}(x;p,q)=p^{n(1-n)/2} \begin{bmatrix}{n}\\{i} \end{bmatrix} _{p,q} p^{i(i-1)/2} x^{i} \bigl((1,x);(p,q)\bigr)_{n-i}, $$

and can be expanded in the basis $\{x^{k}\}_{k \geq0}$ as

6 $$ b_{i}^{n}(x;p,q)=\sum _{k=i}^{n} (-1)^{k-i} q^{(k-i)(k-i-1)/2} p^{\frac{1}{2} ((i-1) i+k (k-2 n+1))} \begin{bmatrix}{n}\\{k} \end{bmatrix} _{p,q} \begin{bmatrix}{k}\\{i} \end{bmatrix} _{p,q} x^{k}. $$

From the definition of $(p,q)$-Bernstein polynomials it is possible to derive the basic properties of $(p,q)$-Bernstein polynomials.

1. Partition of unity
  $$\sum_{i=0}^{n} b_{i}^{n}(x;p,q)=1. $$
2. End-point properties
  $$b_{i}^{n}(0;p,q)= \textstyle\begin{cases} 1, & i=0, \\ 0, & \text{otherwise}, \end{cases}\displaystyle \qquad b_{i}^{n}(1;p,q)= \textstyle\begin{cases} 1, & i=n, \\ 0, & \text{otherwise}. \end{cases} $$

The $(p,q)$-Jacobi polynomials are defined by

7 $$ P_{n}(x;\alpha,\beta;p,q)= {}_{2} \Phi_{1}\left ( \textstyle\begin{array}{c}{(p^{-n},q^{-n}),(p ^{\alpha+\beta+n+1},q^{\alpha +\beta+n+1})}\\ {(p ^{\beta+1},q^{\beta+1})} \end{array}\displaystyle \Big|{(p,q)};{ \frac{x q^{-\alpha}}{p}} \right ) , $$

and they satisfy the second order $(p,q)$-difference equation

8 $$ \begin{aligned}[b] &\frac{q x (q x-p)}{p^{2}} \bigl({ \mathcal{D}}_{p,q}^{2} y \bigr) (x)+ \biggl( \frac {x (p^{\alpha+\beta+2} q^{-\alpha-\beta}-q^{2} )-p^{\beta+2} q^{-\beta}+p q}{p^{2} (p-q)} \biggr) \text{${\mathcal {L}}_{p}$} \bigl((\text{${\mathcal {D}}_{p,q}$}y) (x) \bigr) \\ &\quad+ [n]_{p,q} \biggl(\frac{q p^{-n-2}-p^{\alpha+\beta -1} q^{-\alpha -\beta-n}}{p-q} \biggr) \text{${\mathcal {L}}_{pq}$} y(x)=0. \end{aligned} $$

The $(p,q)$-Jacobi polynomials satisfy the three-term recurrence relation

$$ \begin{gathered} P_{0}(x;\alpha,\beta;p,q)=1, \qquad P_{1}(x;\alpha,\beta ;p,q)=x-B_{0}(\alpha,\beta;p,q), \\ P_{n+1}(x;\alpha,\beta;p,q)= \bigl(x-B_{n}(\alpha,\beta;p,q) \bigr) P_{n}(x;\alpha,\beta;p,q) - C_{n}(\alpha,\beta;p,q) P_{n-1}(x;\alpha,\beta;p,q), \end{gathered} $$

where

9 $$ \begin{aligned}[b] B_{n}(\alpha,\beta;p,q)={}& \frac{p^{n+2} q^{\alpha+n+1}}{(p-q)^{2} [\alpha+\beta+2 n]_{p,q} [\alpha+\beta+2 n+2]_{p,q}} \\ &\times \bigl( \bigl(p^{\beta}+q^{\beta} \bigr) q^{\alpha+\beta+2 n+1}-(p+q) \bigl(p^{\alpha}+q^{\alpha} \bigr) p^{\beta+n} q^{\beta +n} \\ &+ \bigl(p^{\beta}+q^{\beta } \bigr) p^{\alpha+\beta+2 n+1} \bigr) \end{aligned} $$

and

10 $$ C_{n}(\alpha,\beta;p,q)=\frac{p^{\beta+2 n+3} q^{2 \alpha+\beta+2 n+1} [n]_{p,q} [\alpha+n]_{p,q} [\beta +n]_{p,q} [\alpha +\beta+n]_{p,q}}{[\alpha+\beta +2 n-1]_{p,q} ([\alpha+\beta+2 n]_{p,q})^{2} [\alpha+\beta+2 n+1]_{p,q}}. $$

## Representation of $(p,q)$-Bernstein polynomials in terms of $(p,q)$-Jacobi polynomials

### Lemma 3.1

*The* $(p,q)$-*Bernstein polynomials satisfy the following first order* $(p,q)$-*difference equation*:

11 $$ (p x-1) x \bigl(D_{p,q}b_{i}^{n} \bigr) (x;p,q) + \bigl(-p^{1-n} [n]_{p,q} x+p^{-i} [i]_{p,q} \bigr) b_{i}^{n}(p x;p,q) =0. $$

### Proof

The result can be obtained by equating the coefficients in $x^{j}$. □

If we introduce the first order $(p,q)$-difference operator

12 $$ L_{i,n}=(p x-1) x D_{p,q}+ \bigl(-p^{1-n} [n]_{p,q} x+p^{-i} [i]_{p,q} \bigr) {\mathcal{L}}_{p}, $$

then

$$L_{i,n}b_{i}^{n}(x;p,q)=0. $$

### Lemma 3.2

*The* $(p,q)$-*Jacobi polynomials satisfy the following structure relation*:

13 $$ \begin{aligned}[b] &x (p x-1 ) D_{p,q} \bigl(P_{n} \bigl(p^{2} x;\alpha,\beta;p,q \bigr) \bigr) \\ &\quad= [n]_{p,q} p^{-n-2} P_{n+1} \bigl(p^{3}x;\alpha, \beta;p,q \bigr) + \varpi_{1}(n) P_{n} \bigl(p^{3}x; \alpha,\beta;p,q \bigr) \\ &\qquad+ \varpi_{2}(n) P_{n-1} \bigl(p^{3}x;\alpha, \beta;p,q \bigr), \end{aligned} $$

*where*

$$ \begin{gathered} \varpi_{1}(n)=-\frac{[n]_{p,q} (-(p+q) q^{\alpha +n}-p^{\beta +n}+p^{\alpha+\beta+2 n+1}+q^{\alpha+\beta+2 n+1} ) [\alpha +\beta+n+1]_{p,q}}{(p-q) [\alpha+\beta+2 n]_{p,q} [\alpha +\beta+2 n+2]_{p,q}}, \\ \varpi_{2}(n)=\frac{q^{\alpha+n} p^{\beta+2 n+1} [n]_{p,q} [\alpha+n]_{p,q} [\beta+n]_{p,q} [\alpha+\beta+n]_{p,q} [\alpha+\beta+n+1]_{p,q}}{[\alpha+\beta+2 n-1]_{p,q} ([\alpha+\beta+2 n]_{p,q})^{2} [\alpha +\beta+2 n+1]_{p,q}}. \end{gathered} $$

### Proof

The result follows from (7) by equating the coefficients in $x^{j}$. □

### Theorem 3.1

*The* $(p,q)$-*Bernstein polynomials defined in* (5) *have the following representation in terms of* $(p,q)$-*Jacobi polynomials defined in* (7):

14 $$ b_{i}^{n}(x;p,q)=\sum _{k=0}^{n} H_{k}(i,n;\alpha,\beta ;p,q)P_{k} \bigl(p^{2}x;\alpha,\beta;p,q \bigr), $$

*where the connection coefficients* $H_{k}(i,n;\alpha,\beta;p,q)$ *satisfy the following three*-*term recurrence relation*:

15 $$ \begin{aligned}[b] &H_{k-1}(i,n;\alpha, \beta;p,q) \Lambda_{1}(k-1,i,n; \alpha,\beta;p,q)+ H_{k}(i,n; \alpha,\beta;p,q) \Lambda_{2}(k,i,n; \alpha,\beta;p,q) \\ &\quad+ H_{k+1}(i,n;\alpha,\beta;p,q) \Lambda_{3}(k+1,i,n; \alpha, \beta;p,q)=0, \end{aligned} $$

*valid for* $1 \leq k \leq n-1$ *with initial conditions*

16 $$\begin{aligned}& H_{n+1}(i,n;\alpha,\beta;p,q)=0, \end{aligned}$$

17 $$\begin{aligned}& H_{n}(i,n;\alpha,\beta;p,q)=(-1)^{n+1} q^{-\frac{1}{2} (1-n) n} p^{-n (n + 3)/2 + k(k+1)/2} \begin{bmatrix}{n}\\{i} \end{bmatrix} _{p,q}, \end{aligned}$$

*and*

18 $$ \textstyle\begin{cases} \Lambda_{1}(k,i,n;\alpha,\beta;p,q)=p^{-k-2} [k]_{p,q}-p^{-n-2}[n]_{p,q},\\ \Lambda_{2}(k,i,n;\alpha,\beta;p,q)=p^{-i} [i]_{p,q} - p^{-2-n} [n]_{p,q} B_{k}(\alpha,\beta;p,q) + \varpi_{1}(k), \\ \Lambda_{3}(k,i,n;\alpha,\beta;p,q)=-p^{-n-2} [n]_{p,q} C_{k}(\alpha,\beta;p,q) + \varpi_{2}(k). \end{cases} $$

### Proof

In order to obtain the result we shall apply the so-called Navima algorithm (see *e.g.* [19, 20] and the references therein) for solving connection problems. If we apply the first order linear operator $L_{i,n}$ defined in (12) to both sides of (14) we have

$$ \begin{aligned} 0={}&\sum_{k=0}^{n} H_{k}(i,n; \alpha,\beta;p,q)L_{i,n} P_{k} \bigl(p^{2}x;\alpha, \beta;p,q \bigr) \\ ={}&\sum_{k=0}^{n} H_{k}(i,n; \alpha,\beta;p,q) \bigl((px-1) x D_{p,q} \bigl(P_{k} \bigl(p^{2}x;\alpha,\beta;p,q \bigr) \bigr) \\ &+ \bigl(-p^{1-n} [n]_{p,q} x+p^{-i} [i]_{p,q} \bigr) P_{k} \bigl(p^{3}x; \alpha,\beta;p,q \bigr) \bigr). \end{aligned} $$

From the three-term recurrence relation for $(p,q)$-Jacobi polynomials it yields

$$ \begin{gathered} \bigl(-p^{1-n} [n]_{p,q} x+p^{-i} [i]_{p,q} \bigr) P_{k} \bigl(p^{3}x;\alpha,\beta;p,q \bigr) \\ \quad=-p^{-n-2} [n]_{p,q} P_{k+1} \bigl(p^{3}x;\alpha,\beta;p,q \bigr) \\ \qquad{}+ p^{-2-n-i} \bigl( -p^{n+2} [i]_{p,q}+p^{i} [n]_{p,q} B_{k}(\alpha,\beta;p,q) \bigr) P_{k} \bigl(p^{3}x; \alpha,\beta;p,q \bigr) \\ \qquad{}-p^{-n-2} [n]_{p,q} C_{k}(\alpha, \beta;p,q)P_{k-1} \bigl(p^{3}x;\alpha,\beta;p,q \bigr). \end{gathered} $$

Therefore, by using the structure relation for $(p,q)$-Jacobi polynomials (13) we have

$$ \begin{gathered} (px-1) x D_{p,q} \bigl(P_{k} \bigl(p^{2}x;\alpha, \beta;p,q \bigr) \bigr) + \bigl(-p^{1-n} [n]_{p,q} x+p^{-i} [i]_{p,q} \bigr) P_{k} \bigl(p^{3}x;\alpha,\beta;p,q \bigr) \\ \quad= \Lambda_{1}(k,i,n;\alpha,\beta;p,q) P_{k+1} \bigl(p^{3}x;\alpha,\beta;p,q \bigr) +\Lambda_{2}(k,i,n; \alpha,\beta;p,q) P_{k} \bigl(p^{3}x;\alpha,\beta;p,q \bigr) \\ \qquad{}+\Lambda_{3}(k,i,n;\alpha,\beta;p,q) P_{k-1} \bigl(p^{3}x;\alpha,\beta;p,q \bigr), \end{gathered} $$

where $\Lambda_{i}(k,i,n;\alpha,\beta;p,q)$ are given in (18).

As a consequence,

$$ \begin{aligned} 0={}&\sum_{k=0}^{n} H_{k}(i,n; \alpha,\beta;p,q) \bigl(\Lambda_{1}(k,i,n; \alpha,\beta;p,q) P_{k+1} \bigl(p^{3}x;\alpha,\beta;p,q \bigr) \\ &+ \Lambda_{2}(k,i,n;\alpha,\beta;p,q) P_{k} \bigl(p^{3}x;\alpha,\beta;p,q \bigr) + \Lambda_{3}(k,i,n; \alpha,\beta;p,q) P_{k-1} \bigl(p^{3}x;\alpha,\beta;p,q \bigr) \bigr). \end{aligned} $$

By using the linear independence of $\{P_{k}(p^{3}x;\alpha,\beta ;p,q)\}$ we obtain the three-term recurrence relation (15) for the connection coefficients $H_{k}(i,n;\alpha,\beta;p,q)$, where the initial conditions are obtained by equating the highest power in $x^{k}$. □

## Conclusions

In this work we have obtained a three-term recurrence relation for the coefficients in the expansion of $(p,q)$-Bernstein polynomials in terms of $(p,q)$-Jacobi polynomials. For our purposes some auxiliary results both for $(p,q)$-Bernstein polynomials and $(p,q)$-Jacobi polynomials have been derived.
